# Bone Marrow-Derived Microglia Infiltrate into the Paraventricular Nucleus of Chronic Psychological Stress-Loaded Mice

**DOI:** 10.1371/journal.pone.0081744

**Published:** 2013-11-26

**Authors:** Koji Ataka, Akihiro Asakawa, Kanna Nagaishi, Kaori Kaimoto, Atsushi Sawada, Yuko Hayakawa, Ryota Tatezawa, Akio Inui, Mineko Fujimiya

**Affiliations:** 1 Department of Anatomy, Sapporo Medical University School of Medicine, Sapporo, Japan; 2 Department of Psychosomatic Internal Medicine, Kagoshima University Graduate School of Medical and Dental Sciences, Kagoshima, Japan; 3 Department of Anesthesiology, Sapporo Medical University School of Medicine, Sapporo, Japan; University of Florida, United States of America

## Abstract

**Background:**

Microglia of the central nervous system act as sentinels and rapidly react to infection or inflammation. The pathophysiological role of bone marrow-derived microglia is of particular interest because they affect neurodegenerative disorders and neuropathic pain. The hypothesis of the current study is that chronic psychological stress (chronic PS) induces the infiltration of bone marrow-derived microglia into hypothalamus by means of chemokine axes in brain and bone marrow.

**Methods and Findings:**

Here we show that bone marrow-derived microglia specifically infiltrate the paraventricular nucleus (PVN) of mice that received chronic PS. Bone marrow derived-microglia are CX_3_CR1^low^CCR2^+^CXCR4^high^, as distinct from CX_3_CR1^high^CCR2^-^CXCR4^low^ resident microglia, and express higher levels of interleukin-1β (IL-1β) but lower levels of tumor necrosis factor-α (TNF-α). Chronic PS stimulates the expression of monocyte chemotactic protein-1 (MCP-1) in PVN neurons, reduces stromal cell-derived factor-1 (SDF-1) in the bone marrow and increases the frequency of CXCR4^+^ monocytes in peripheral circulation. And then a chemokine (C-C motif) receptor 2 (CCR2) or a β_3_-adrenoceptor blockade prevents infiltration of bone marrow-derived microglia in the PVN.

**Conclusion:**

Chronic PS induces the infiltration of bone marrow-derived microglia into PVN, and it is conceivable that the MCP-1/CCR2 axis in PVN and the SDF-1/CXCR4 axis in bone marrow are involved in this mechanism.

## Introduction

Microglia are innate immune-defense cells that react to brain infection and inflammation. During the embryonic stage, resident microglia migrate from the yolk sac into the brain where they reside for life [1,2]. Recently bone marrow-derived microglia have been reported to infiltrate into the brain parenchyma from the blood during brain injury, amyotrophic lateral sclerosis (ALS), multiple sclerosis, experimental autoimmune encephalomyelitis (EAE), and Alzheimer’s disease [3–9]. 

The recruitment of bone marrow-derived cells into the brain in functional disorders caused by stress has not been well studied. We recently reported that chronically repeated foot-shock stress induced a massive infiltration of bone marrow-derived cells into the ventral hippocampus in mice [10]. However, to our knowledge, no studies have examined whether psychological stress can induce such infiltration into the CNS. Psychological stress originating from various somatosensory and nociceptive inputs is processed through higher centers of the brain and influences learning, emotional, and cognitive functions [11]. It is strongly related to anxiety, depression, and functional gastrointestinal disorders (FGIDs) including irritable bowel syndrome (IBS), functional dyspepsia (FD), and eating disorders [12,13]. 

The communication box (CB) method used in the present study is an experimental model to expose animals to psychological stress through visual, auditory, and olfactory stimuli produced by neighboring animals that receive electrical foot-shocks (Figure S1A). Psychological stress induced by CB has previously been shown to cause food intake suppression, anxiety, and depression [14–16]. We also recently demonstrated that chronic psychological stress (chronic PS) induced by CB decreases antral motility and increases colonic motility in mice, which mimics FD and IBS [17]. 

The present study investigated the effects of chronic PS on the interaction between bone marrow-derived microglia and neurons, which has until now only been examined in the case of injury, inflammation, or neurodegenerative disease. Mechanisms for the recruitment of monocytes from bone marrow into the peripheral circulation and subsequent migration into specific brain nuclei were also examined. This study may give perspectives for the neuroregulatory effects of microglia in psychological stress reactions.

## Methods

### Animals

C57BL/6 male mice weighing 20–25 g at the start of the experiments were maintained under conditions of controlled temperature (22–24 °C), humidity (44–46%), and a 12-h light/dark cycle (light on 7:00–19:00). Food and water were available *ad libitum*. Mice were used once for each experiment. All animal experiments were approved by the Institutional Animal Care and Use Committee at Sapporo Medical University School of Medicine, Sapporo, Japan. We isolated bone marrow cells from tibias and femurs of adult GFP transgenic mice (C57BL/6 –Tg(CAG-EGFP), Japan SLC). These C57BL/6 EGFP transgenic mice have expression of enhanced green fluorescent protein (EGFP) directed to widespread tissues by the CMV-IE enhancer/chicken β-actin/rabbit β-globin hybrid promoter. Bone marrow cells (1 × 10^5^ cells) were injected into the tail vain of recipient mice, which received whole-body irradiation of 9 Gy. To evaluate the effect of irradiation on infiltration of bone marrow-derived cells into brain, recipient mice were covered with a lead cap and received irradiation of 9 Gy. Transplanted recipient mice were maintained in cages covered by filter caps and given sterile water including 0.001 N HCl (pH 2.0) and sterile chow for two weeks to prevent infection. Eight to ten weeks after the bone marrow transplantation, the ratio of GFP positive cells in monocytes were examined in each mouse by FACS. Mice with chimeric ratio of larger than 90% were used in this study (Figure S1B and C). No difference was found in chimeric rates between mice with whole body irradiation and specific body irradiation with head protection.

### Exposure to chronic PS by the CB

Eight to ten weeks after the bone marrow transplantation, mice were handled daily for 10 min by the same investigator for at least one week to prevent stress caused by subsequent experimental handling. The CB consists of nine compartments divided by transparent acrylic panels (Figure S1A; 16 × 16 × 40 cm, BS-CC01; BrainScience-idea, Osaka, Japan). Five electrical foot shock (FS) compartments have a grid floor made of stainless steel rods connected to an electric generator (BS-5ES; BrainScience-idea) and four compartments have a safety grid floor with no electrical connection. Five mice were placed individually in each of the FS compartments and received a 0.2 mA electric current of 10 s duration delivered randomly an average of twice per min for 60 min. Four more mice were placed individually in the psychological stress (PS) compartments with the safety floor. Mice in FS compartments cry and jump during 10s of electrical FS, and evacuate their bowels. Mice in PS compartments were surrounded by FS compartments on three sides, received visual, auditory, and olfactory stimuli from mice receiving electrical FS (Figure S1A). CB stress stimulation was performed for 1 h (10:00–11:00) daily and continued for five successive days, because we previously observed abnormal gastrointestinal motility caused by CB stimulation at fifth day of PS procedure [17]. Sham-treated controls were placed in PS compartments similar to the experimental group but with no stimuli. 

### Quantification of microglia infiltrating in the CNS

Immediately after stress-loading, mice were anesthetized with i.p. injection of sodium pentobarbital (50 mg/kg) and perfused through the left ventricle of the heart with phosphate-buffered saline (PBS), then 4% paraformaldehyde at the flow rate of 3 ml/min. Brains were cut into serial 20 µm coronal sections in a cryostat. We counted the number of GFP-positive cells at one side of the PVN in sections cut through hypothalamus at 200× magnification under confocal laser microscopy (A1; Nikon, Japan). The maximum number of GFP^+^ cells from one section was obtained from each animal and used for analysis. PVN were distinguished according to Mouse Brain in Stereotaxic Coordinates written by Franklin & Paxinos.

### Immunohistochemistry

Brain sections were incubated with a primary antibody for one to two days at a dilution of 1:500 for Ionized calcium-binding adapter molecule 1 (Iba-1, 019-19745; Wako Pure Chemical Industries, Osaka, Japan), 1:200 for Glial fibrillary acidic protein (GFAP, AB5541; Millipore, Billerica, CA), 1:100 for Monocyte Chemotactic Protein-1 ( MCP-1, ab7202; Abcam, Boston, MA or SC-1784; Santa Cruz, Dallas TX), 1:100 for phosphorylated N-methyl-D-aspartate receptor (pNMDAR, 04-1064; Millipore), 1:100 for interleukin-1β (IL-1β, 503501; BioLegend), 1:250 for Protein gene product 9.5 (PGP9.5, AB5898, Millipore), 1:250 for NeuN (Neuronal Nuclei, ABN78; Millipore), or 1:25 for IL-1 receptor (AF771; R&D). Sections were then incubated for 2 h with Cy3, Cy5 or Alexa Fluoro 647-conjugated secondary antibody diluted 1:500. Nuclei were counterstained with 4',6-Diamidino-2-phenylindole dihydrochloride solution (DAPI, D523; Dojindo, Kumamoto, Japan). The image was observed using confocal laser microscopy (Nikon A1). In the quantification of MCP-1^+^NeuN^+^ cells or MCP-1^+^GFAP^+^ cells in PVN, we counted the number of MCP-1^+^NeuN^+^ cells or MCP-1^+^GFAP^+^ cells at one side of the PVN in sections cut through hypothalamus at 400× magnification under confocal laser microscopy (A1; Nikon). The maximum number of GFP^+^ and Iba-1^+^ cells from one section was obtained from each animal and used for analysis.

### Isolation of microglia

Mice were anesthetized with i.p. injection of sodium pentobarbital (50 mg/kg) and perfused with sterile 0.1 M PBS for 5 min at the flow rate of 3 ml/min immediately after PS exposure on day 5. Hypothalamic tissues taken from four mice were put together (*n* = 1) and dissociated to single-cell suspensions using the Neural Tissue Dissociation Kit (130-092-628; Miltenyi Biotec Inc., Germany) and Gentle MACS Dissociator (130-093-235; Miltenyi Biotec Inc.). Cells were washed with MACS buffer, and treated with MACS buffer containing FcR blocker (130-092-577; Miltenyi Biotec Inc.) then CD11b (microglia) MicroBeads (130-093-634; Miltenyi Biotec Inc.). CD11b-positive cells were isolated with a MACS MS column (130-042-201; Miltenyi Biotec Inc.), stained with anti-CD45 conjugated to APC (559864; BD Biosciences, Sparks, MD) and sorted with FACS Aria II (BD Biosciences). We used 12 mice to obtain the data (*n* = 4). The numbers of cells per 20000 total events in gate (1) or (2) were counted by FACS.

### Quantitative real-time RT-PCR analysis

Total RNA was extracted from sorted microglia or isolated hypothalamus tissues using the RNeasy Micro Kit (74004; Qiagen, Hilden, Germany). cDNA was synthesized using SuperScript III First-Strand Synthesis System for RT-PCR (18080-051; Invitrogen, Carlsbad, CA) according to the manufacturer’s instructions. Quantitative RT-PCR for the expression of CCR2, CX_3_CR1, CXCR4, excitatory amino acid transporter 1(EAAT1), EAAT2, purinergic receptors (P2X4, P2X7, P2Y1, and P2Y12), IL-1β and tumor necrosis factor-α (TNF-α) on isolated microglia, and for the expression of MCP-1, stromal cell-derived factor 1(SDF-1), and fractalkine in hypothalamic tissues was performed on the ABI prism 7500 Sequence Detection System (Applied Biosystems, Foster City, CA) with Power SYBAR GREEN PCR Master Mix (4367659; Applied Biosystems). Relative mRNA expression was quantified by the 2^-∆CT^ method. Primer sequences are shown in Table S1. 

### The Length of axis of bone marrow-derived microglia

We measured the length of axis of GFP^+^Iba-1^+^ (bone marrow-derived microglia, BMDM) and GFP^−^Iba-1^+^ cells (resident microglia, RM) in chronic psychological stress-loaded and sham mice. The image was observed, and the length was measured using confocal laser microscopy and NIS Elements analysis software (Nikon). 

### Quantification of GFP^+^CCR2^+^ cells in peripheral blood and hypothalamus

 Peripheral blood was sampled from chronic PS-loaded mice received the transplantation of bone marrow cells from GFP transgenic mice, and stained with anti-CCR2 antibody (ab32144, abcam) following to Alexa Fluor 647 conjugated secondary antibody (711-605-152, Jackson). The frequency was calculated from the following formula: the number of cells in the gate of GFP^+^CCR2^+^ divided by the number of cells in monocyte area gated by SSC-A and FFC-A on FACS. To count the number of CCR2^+^ cells in hypothalamus of chronic PS-loaded and sham-treated mice, isolated CD11b^+^ cells as mention above were stained with anti-CCR2 antibody followed by PE conjugated secondary antibody (12-4739, eBioscience, San Diego, CA), and then the numbers of GFP^+^CCR2^+^ and GFP^−^CCR2^+^cells per 10000 total events were counted by FACS.

### Quantification of SDF-1 in bone marrow and GFP^+^CXCR4^+^ cells in peripheral blood

Femora from chronic PS-loaded mice without the irradiation were flushed out with Hanks’ balanced salt solution. Cells were removed by centrifugation and the supernatant was assayed with an SDF-1 ELISA kit (C06021188, RayBiotech, Norcross, GA). Peripheral blood was obtained from chronic PS-loaded and sham-treated mice that received the transplantation of bone marrow cells from GFP transgenic mice. The blood samples were hemolyzed with RBC Lysis buffer (1045695, QIAGEN) and stained with anti-CXCR4 conjugated to APC (558644; BD Biosciences). The frequency was calculated from the following formula: the number of cells included in the gate of CXCR4^+^ area divided by the number of cells in the monocyte area gated by side scatter pulse-area (SSC-A) and forward scatter pulse-area (FFC-A) on FACS.

### The effects of antagonists on the accumulation of bone marrow-derived microglia

A CCR2 antagonist, RS102895 (R1903; Sigma, St Louis, MO), was administered orally with gavage (5 mg/kg), or a β_3_-adrenergic blocker, SR59230A (1511; Tocris, Bristol, UK), was i.p. injected (1-mg/kg) daily 30 min before the PS stimulation. The sections of brain were prepared and stain with Iba-1 antibody following to Cy3-conjugated secondary antibody. We counted the number of GFP-positive cells at one side of the PVN in sections cut through hypothalamus at 200× magnification under confocal laser microscopy (A1; Nikon). The maximum number of GFP^+^ and Iba-1^+^ cells from one section was obtained from each animal and used for analysis. 

### Measurement of anxiety-like behavior

To evaluate the anxiety-like behavior in chronic PS-loaded mice we used the elevated plus-maze test according to the previous study [18]. Mice without irradiation and also without chimerism were administrated RS102895 (5 mg/kg) or saline (200 µl) orally with gavage daily 30 min before the psychological stress stimulation. After chronic psychological stress procedure on day 5 mice were put on the center of the elevated plus-maze apparatus and freely explore the maze for 5 min. We measured the time spent in open arms. 

### Statistical analysis

Data are expressed as means ± sem. Comparisons between two groups were performed using the two-tailed Student’s *t*-test, while one-way analysis of variance (ANOVA) followed by Tukey’s Multiple Comparison Test were used to compare groups of three or more. Differences were considered significant at *P* < 0.05.

## Results

### Chronic PS induces the infiltration of bone marrow-derived microglia into PVN of the hypothalamus

**Figure 1:**
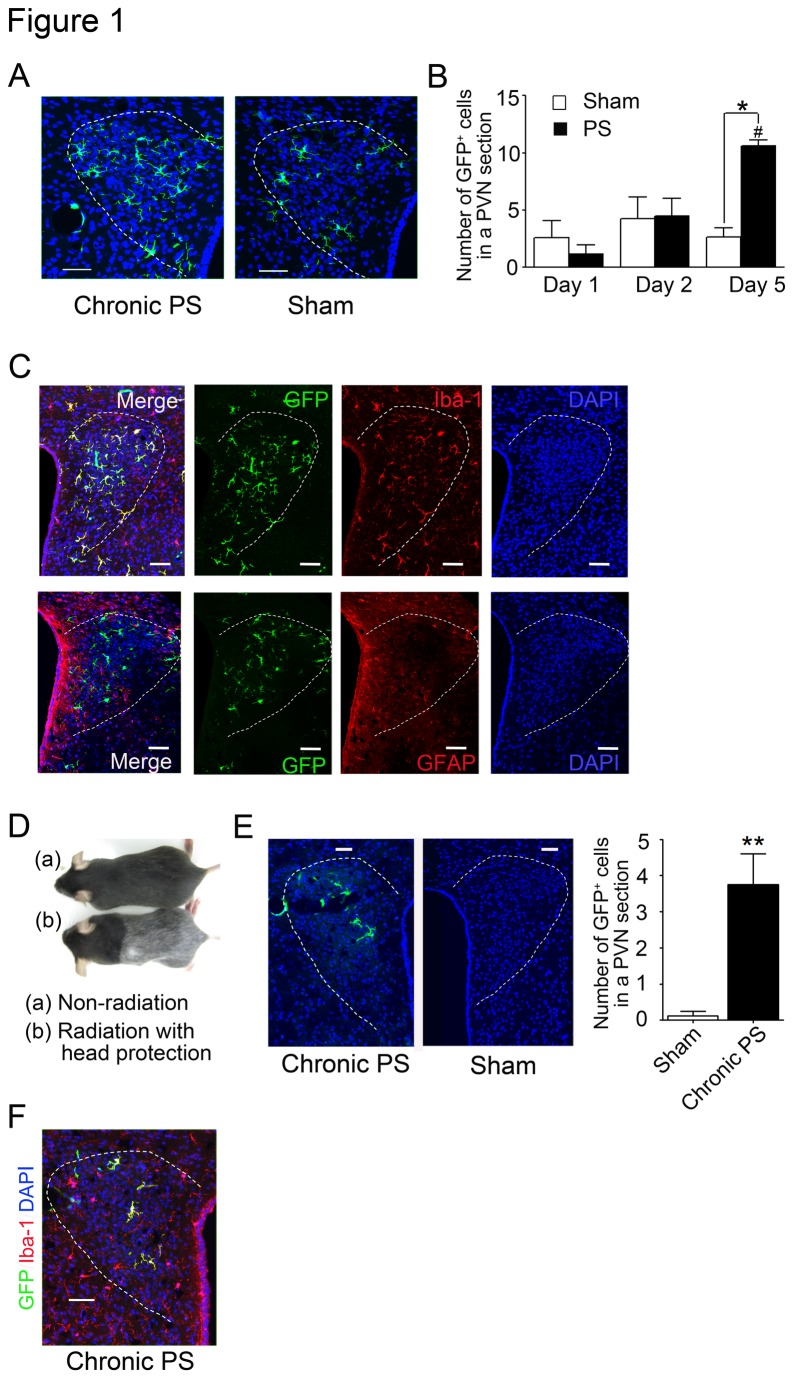
Chronic psychological stress (PS) increases the infiltration of bone marrow-derived microglia into the PVN. (A, B) After chronic PS-loading for five days, the numbers of GFP-positive cells (green) in the PVN were significantly increased compared with sham-treated mice. Data are expressed as mean ± sem (PS: *n* = 6−7, sham: *n* = 5−11). ***#*** *P* < 0.05 with ANOVA followed by Tukey’s multiple comparison. *P < 0.05 with two-tailed Student’s *t*-test. (C) GFP-positive cells (green) overlapped with Iba-1 (red) in PVN from chronic PS-loaded mice but did not overlap with GFAP (red) in PVN from chronic PS-loaded mice. (D) Photograph of mice received irradiation with head protection and non-irradiated mice. (E, F) After chronic PS-loading for five days, the number of GFP-positive cells (green) in the PVN of mice received the irradiation with head protection was significantly increased compared with sham-treated mice. Data are expressed as mean ± sem. **P < 0.001 for PS (*n* = 8) to sham (*n* = 4) with two-tailed Student’s *t*-test. (G) GFP-positive cells (green) overlapped with Iba-1 (red) in PVN from chronic PS-loaded mice received the irradiation with head protection. PVN shown by dotted line. Scale bars: 50 µm.

The number of GFP-positive cells in the PVN of PS-loaded mice was 1.2 ± 0.8 on day 1 and 4.5 ± 1.1 on day 2, which were not significantly different from those of sham-treated mice (2.6 ± 1.5 on day 1 and 4.3 ± 1.9 on day 2). On day 5, however, GFP-positive cells were significantly higher in number in PS-loaded mice (10.6 ± 0.5) compared with sham-treated mice (Figure 1A and B; F_5,36_ = 4.43, *P* = 0.01). GFP-positive cells in both chronic PS-loaded and sham-treated mice showed a ramified shape (Figure 1A), and were positively stained with Iba-1 but not GFAP (Figure 1C upper panels). GFP/Iba-1-double positive cells accumulated in the PVN, while GFP-negative/Iba-1-positive cells were diffusely scattered in the brain (Figure 1C lower panels). To evaluate the effects of whole body radiation on infiltration of bone marrow-derived cells, mice were received the specific body irradiation with head protection and bone marrow transplantation from GFP-Tg donors (Figure 1D). Eight to ten weeks after the bone marrow transplantation mice were exposed to psychological stress for five successive days using the CB. GFP-positive cells were increased in the PVN of psychological stress-loaded mice (Figure 1E; 3.8 ± 0.9 in stress-loaded mice and 0.1 ± 0.1 in sham mice, *P* = 0.0022), and stained with Iba-1 antibody (Figure 1F). 

No difference was found in the number of GFP^+^ cells in other area of brain between chronic PS and sham mice (Figure S2 and S3). 

### Bone marrow-derived microglia showed different mRNA expression from resident microglia

**Figure 2:**
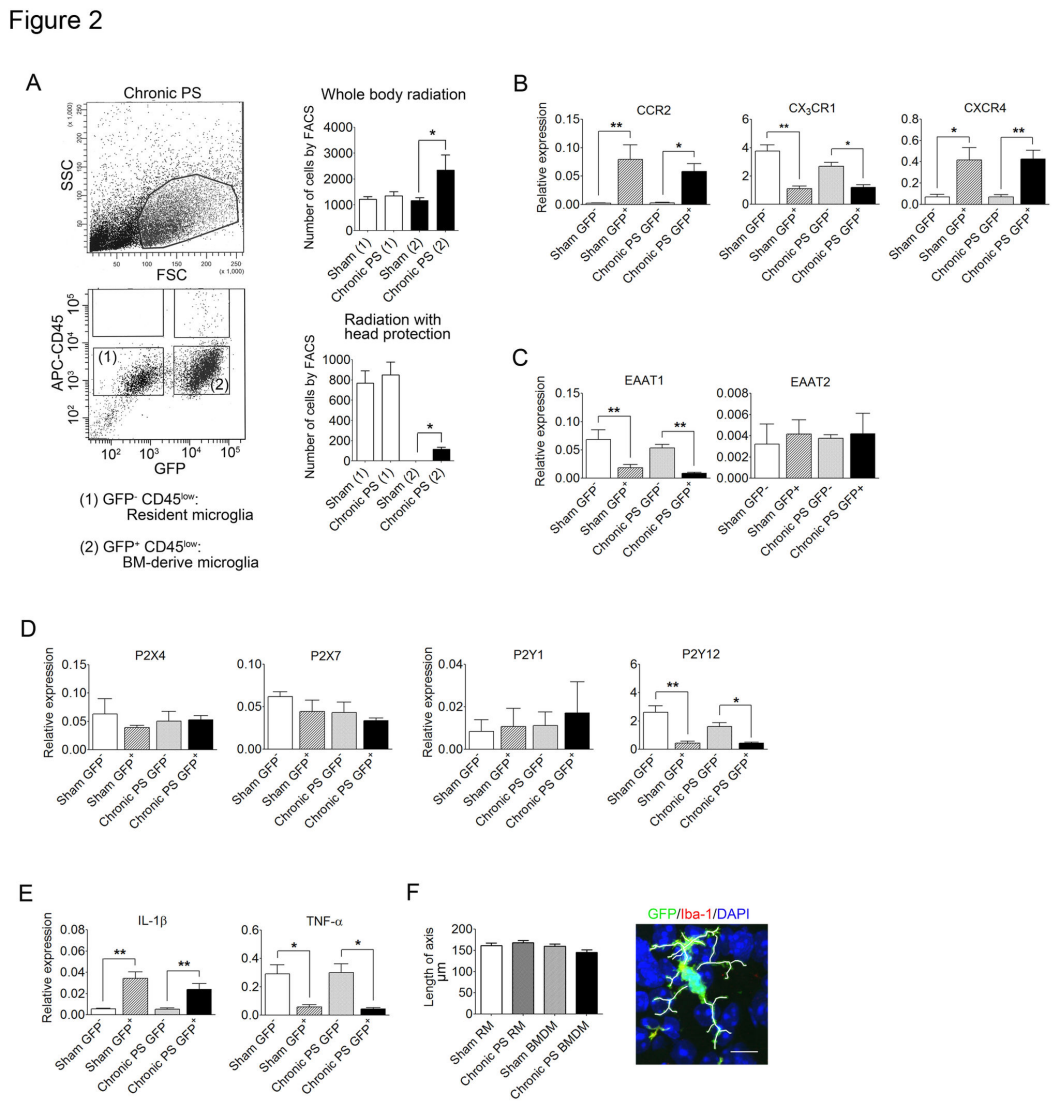
Isolation of bone marrow-derived microglia and resident microglia from hypothalamic tissue and comparison of expression of various molecules in chronic PS-loaded and sham-treated mice. (A) Representative FACS chart in chronic PS-loaded mice with whole body radiation and the number of isolated GFP^-^CD45^low^ (resident microglia) and GFP^+^CD45^low^ (bone marrow-derived microglia) from mice with whole body radiation and the radiation with head protection (*n* = 4−6). Total events in FACS were 20000. Data are expressed as mean ± sem. *P < 0.05 with two-tailed Student’s t-test. (B−E) mRNA expression of chemokine receptors: CCR2 (*n* = 6−8), CX_3_CR1 (*n* = 3−5), CXCR4 (*n* = 3−5) (B), exiting amino acid transporters: EAAT1 (*n* = 4−5), EAAT2 (*n* = 3−4) (C), purinergic P2X receptors: P2X4 (*n* = 3) and P2X7 (*n* = 3) and P2Y receptors: P2Y1 (*n* = 3), P2Y12 (*n* = 3−4) (D), IL-1β (*n* = 4−6) and TNF-α (*n* = 3−5) (E). (F) The length of axis of GFP^+^Iba-1^+^ microglia (bone marrow-derived microglia, BMDM) and GFP^-^Iba-1^+^ microglia (resident microglia. RM) in chronic PS-loaded and sham mice (*n* = 4). Scale bars: 10 µm. Data are expressed as mean ± sem. *P < 0.05, **P < 0.01 with ANOVA followed by Tukey’s multiple comparison.

To avoid contamination of CD11b^+^CD45^+^ macrophages and monocytes, we sorted CD11b^+^CD45^low^ cells to isolate microglia [19] (Figure 2A). The number of GFP^+^CD45^low^ cells was increased in chronic PS-loaded mice compared to sham-treated mice in both whole body radiation and radiation with head protection (Table 1, Figure 2A; P = 0.0042 and < 0.0001, respectively). There was no difference in the number of GFP^−^CD45^low^ cells between chronic PS-loaded and sham-treated mice in both whole body radiation and radiation with head protection (Figure 2A). 

**Table 1:** The number of GFP^-^CD45^low^ and GFP^*+*^CD45^low^ cells.

Group (gate no.)	Whole radiation	Radiation with head protection
Sham (1)	1210 ± 111	768 ± 122
Chronic PS (1)	1342 ± 110	849 ± 126
Sham (2)	1165 ± 110	1 ± 1
Chronic PS (2)	2339 ± 564*	115 ± 20*

* *P* < 0.05 v.s. Sham (2) (*n* = 4−6)

(1): GFP**^*−*^**CD45^low^ cells, (2): GFP**^*+*^**CD45^low^ cells

We analyzed mRNA expression of proteins on GFP^+^CD45^low^ and GFP^−^CD45^low^ cells between chronic PS-loaded and sham-treated mice. No change was observed in the expression of any mRNA studied between chronic PS-loaded and sham-treated mice. However, a significant increase was detected in the mRNA expression of chemokine receptors, including CCR2 and CXCR4, in GFP-positive (bone marrow-derived) microglia compared with GFP-negative (resident) microglia in both chronic PS-loaded and sham-treated mice (Figure 2B; F_3,25_ = 8.676, *P* < 0.0001 and F_3,14_ = 10.68, *P* = 0.0006, respectively). The expression of IL-1β was also increased in GFP-positive microglia compared with GFP-negative microglia in both groups (Figure 2E; F_3,17_ = 15.90, *P* < 0.0001).

On the other hand, the expression of receptor CX_3_CR1, EAAT1 and P2Y12 was decreased in GFP-positive microglia compared with GFP-negative microglia (Figure 2B, C, and D; F_3,12_ = 21.97, *P* < 0.0001, F_3,14_ = 10.21, *P* = 0.0008, and F_3,10_ = 15.68, *P* = 0.0004, respectively). TNF-α expression was also significantly decreased in GFP-positive microglia compared with GFP-negative microglia in both groups (Figure 2E; F_3,12_ = 7.573, *P* = 0.0042). 

To evaluate the morphological differences between bone marrow-derived microglia and resident microglia, the length of axis of those cells was measured. No difference was found in morphology between bone marrow-derived and resident microglia (Figure 2 F).

### Bone marrow-derived cells infiltrate the PVN through MCP-1/CCR2 chemotaxis in chronic PS-loaded mice

**Figure 3:**
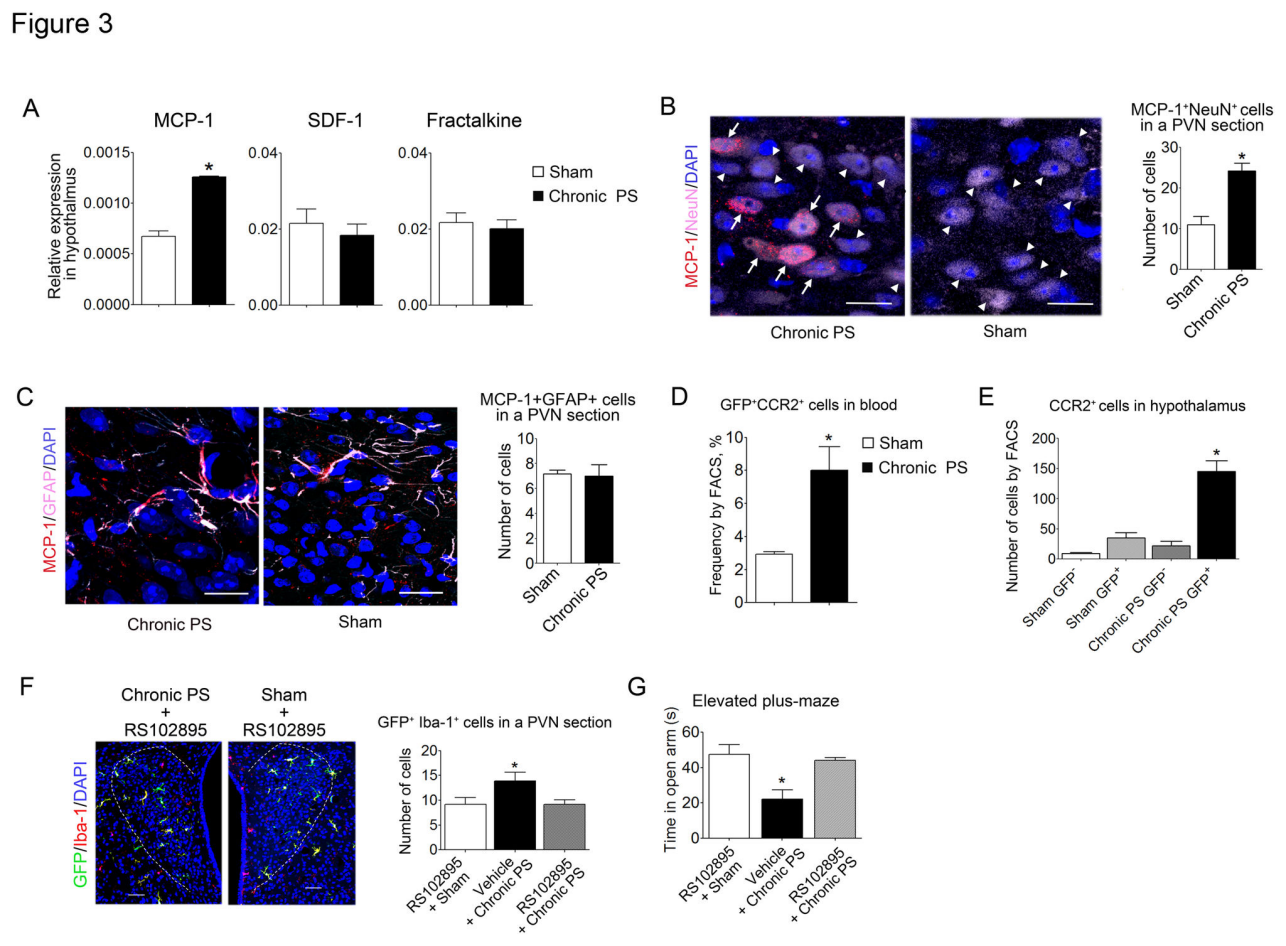
MCP-1/CCR2 axis in hypothalamus and peripheral blood, and effects of CCR2 blockade on the infiltration of bone marrow-derived microglia into the PVN and anxiety-like behavior induced by chronic PS. (**A**) mRNA expression of chemokines in hypothalamic tissue from chronic PS-loaded and sham-treated mice (*n* = 4). Data are expressed as mean ± sem. *P < 0.05 with two-tailed Student’s *t*-test. (B) Immunofluorescence staining with MCP-1 (red) and NeuN (pink) in PVN from chronic PS-loaded and sham-treated mice. Arrows indicate MCP-1^+^NeuN^+^ cells and arrow heads indicate MCP-1^-^NeuN^+^ cells. Scale bars: 20 µm. Data are expressed as mean ± sem (n = 7). *P < 0.05 with two-tailed Student’s *t*-test. (C) Immunofluorescence staining with MCP-1 (red) and GFAP (pink) in PVN from chronic PS-loaded and sham-treated mice. Scale bars: 20µm. Data are expressed as mean ± sem (n = 4−6). *P < 0.05 with two-tailed Student’s t-test. (D) Frequency of GFP^+^CCR2^+^ cells in the peripheral blood obtained by FACS (*n* = 4−6). Data are expressed as mean ± sem. *P < 0.05 with two-tailed Student’s *t*-test. (E) The number of CCR2^+^ cells in the hypothalamus obtained by FACS (*n* = 4). Total events in FACS were 20000. Data are expressed as mean ± sem. *P < 0.05 with ANOVA followed by Tukey’s multiple comparison. (F) Effects of a CCR2 antagonist, RS102895, on infiltration of bone marrow-derived microglia in PVN. PVN is shown by dotted line. Scale bars: 50 µm. Number of GFP-positive cells in a section including PVN is shown. (G) Effect of RS102895 on the anxiety-like behavior induced by chronic PS. Data are expressed as mean ± sem. *P < 0.05 with ANOVA followed by Tukey’s multiple comparison (*n* = 4).

Because bone marrow-derived microglia highly express CCR2, we investigated whether MCP-1/CCR2 axis in brain is involved in the accumulation of bone marrow-derived cells in the PVN. The mRNA expression of MCP-1 in the hypothalamus was increased in chronic PS-loaded mice compared with sham-treated mice, although expression of SDF-1 and fractalkine (a CX_3_CR1 ligand) in the hypothalamus was unchanged between the two groups (Figure 3A; P = 0.0046).

 Increased expression of MCP-1 in the hypothalamus was confirmed by an immunohistochemical study. MCP-1 positive reaction was detected in both NeuN^+^ neurons and GFAP^+^ astrocytes in the PVN (Figure 3 B and C). The number of MCP-1^+^NeuN^+^ cells in the PVN was increased in chronic PS-loaded mice (24.1 ± 1.8) compared to sham-treated mice (10.7 ± 2.1, Figure 3B; P = 0.0005), while the number of MCP-1^+^GFAP^+^ cells in the PVN was unchanged between chronic PS-loaded mice (7.5 ± 0.5) and sham-treated mice (7.0 ± 0.5, Figure 3C). 

In chronic PS-loaded mice the frequency of GFP^+^CCR2^+^ cells was increased in peripheral blood compared to sham-treated mice (Figure 3D, P = 0.0216). On the FACS analysis the number of GFP^+^CCR2^+^ in hypothalamus was increased in chronic PS-loaded mice compared to sham-treated mice (Figure 3 E; F_3,13_ = 30.69, *P* < 0.05). RS 102895 suppressed the accumulation of GFP-positive cells in the PVN induced by chronic PS (Figure 3F, F_2,10_ = 12.45, *P* < 0.0019). Furthermore, in measurement of anxiety-like behavior using the elevated plus-maze methodology on mice without irradiation as well as without bone marrow transplantation, RS102895 reversed the decrease in the time spent in open arms induced by chronic PS to the normal levels (Figure 3G; F_2,9_ = 9.28, *P* = 0.0065). 

### Bone marrow-derived cells egress from bone marrow into peripheral circulation through SDF-1/CXCR4 axis and β_3_-adrenergic mechanisms in chronic PS-loaded mice

**Figure 4:**
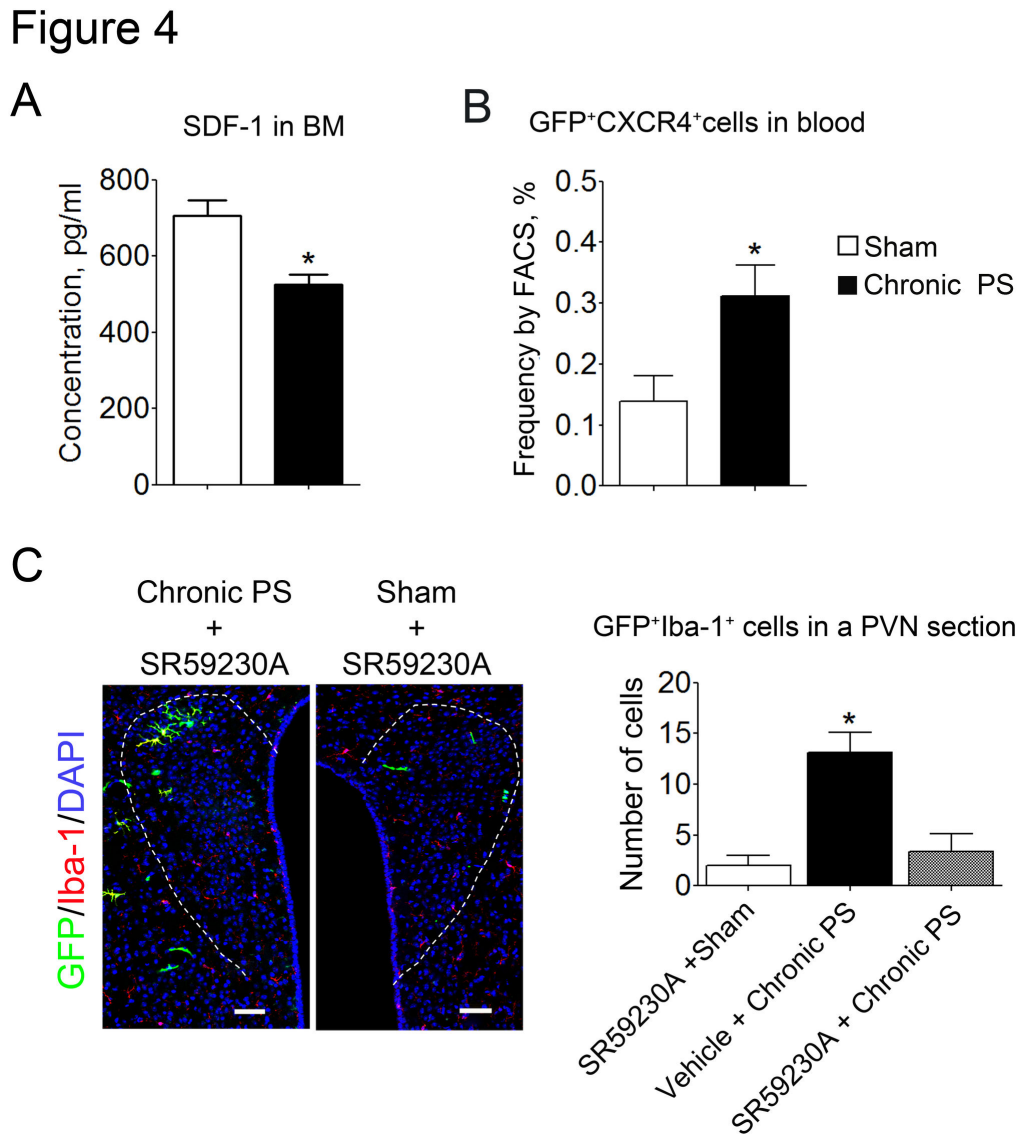
Expression of SDF-1 in the bone marrow and frequency of GFP^+^CXCR4^+^ cells in peripheral blood. Effects of β_3_-adrenergic blockade on the infiltration of bone marrow-derived microglia in the PVN. (A) Chronic PS decreased the SDF-1 concentration in the bone marrow (*n* = 3−4) and increased the frequency of CXCR4^+^ monocytes in the peripheral blood (*n* = 4−6). (B, C) β_3_-adrenoceptor antagonist SR59230A blocks the infiltration of bone marrow-derived microglia in the PVN. Number of GFP-positive cells was counted in a section including PVN. Data are expressed as mean ± sem. *P < 0.05 (*n* = 3−11). PVN shown by dotted line. Scale bars: 50 µm.

Because bone marrow-derived microglia highly express CXCR4, we investigated whether SDF-1/CXCR4 axis in the bone marrow is involved in the recruitment of bone marrow-derived cells in the peripheral circulation. The SDF-1 level in the bone marrow of chronic PS-loaded mice was significantly lower than in sham-treated mice (Figure 4A; P = 0.0126). Moreover, the percentage of GFP-positive CXCR4^+^ peripheral blood monocytes was significantly higher in chronic PS-loaded mice compared with sham-treated mice (Figure 4B; P = 0.0320).

To examine the involvement of β_3_-adrenergic mechanisms in the pathways between chronic PS and the recruitment of bone marrow-derived cells from the bone marrow into the hypothalamus through peripheral blood, we administered SR59230A as a pretreatment. The SR59230A blocked the aggregation of GFP-positive cells in the PVN induced by chronic PS (Figure 4C; F_3,22_ = 6.137, *P* = 0.0034). 

### Bone marrow-derived microglia are IL-1β positive cells and exist in close vicinity to pNMDAR and IL-1 receptor positive neurons

**Figure 5:**
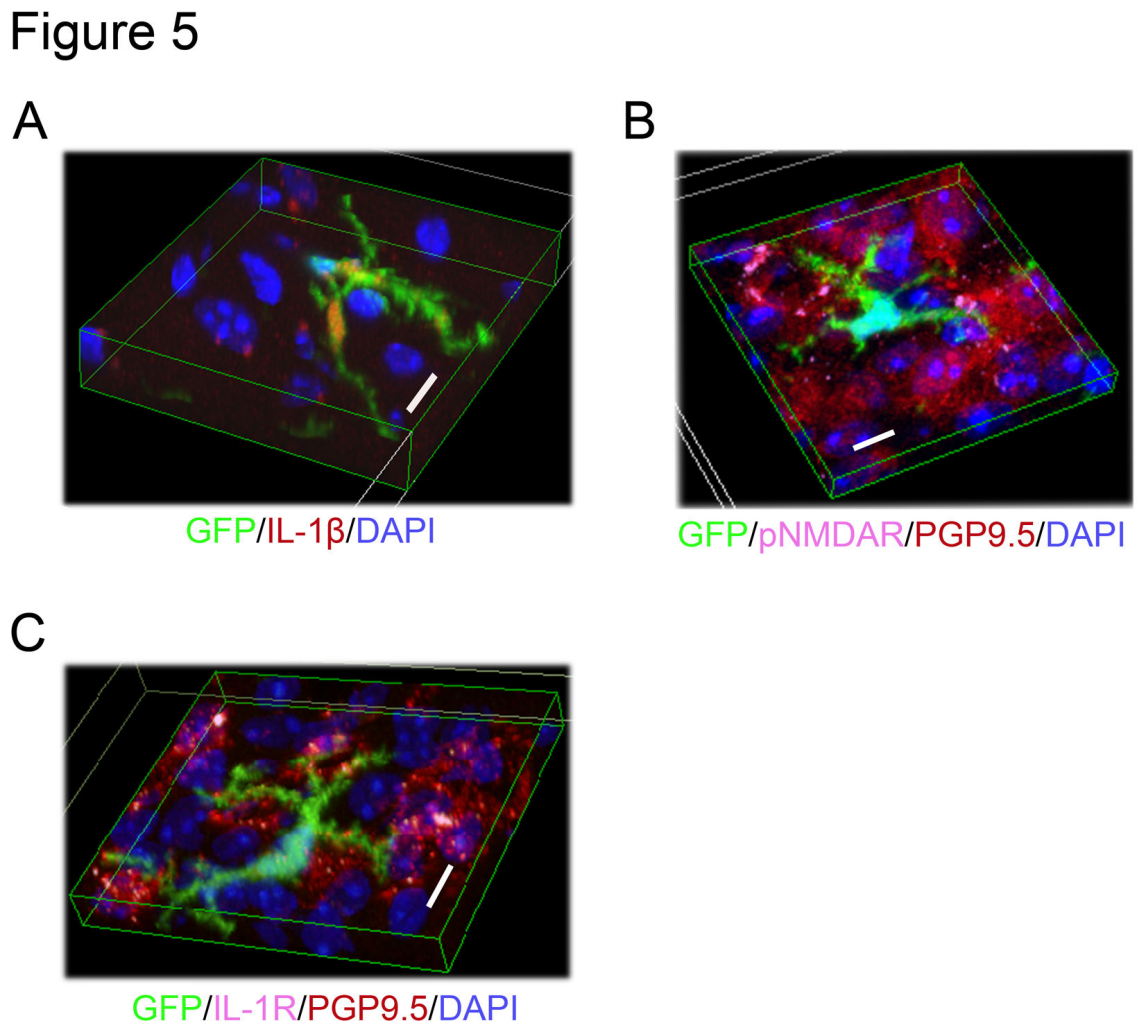
Three dimensional image of immunofluorescence staining of IL-1β, pNMDA receptor, and IL-1 receptor in PVN from chronic PS-loaded mouse. (A) GFP-positive cells (green) overlapped with IL-1β (red). (B, C) Positive reactions for pNMDAR (pink) and IL-1R (pink) detected on the membrane of neurons (red) adjacent to bone marrow-derived microglia (green) in PVN. Scale bars: 10 µm.

By immunhistochemical overlap staining, IL-1β was stained in GFP^+^ cells in the PVN from chronic psychological stress-loaded mice (Figure 5A). Those GFP^+^ cells were located adjacent to pNMDAR positive (Figure 5B) and IL-1 receptor (IL-R) positive neurons (Figure 5C). 

## Discussion

Repeated exposure of PS to mice induces the recruitment of bone marrow derived-microglia into the PVN, which is an important locus for stress-induced functional disorders [20,21]. The number of GFP positive cells in PVN was increased in mice received whole body irradiation compared to mice received specific body irradiation with head protection, indicating that irradiation affected the permeability of BBB. In fact, in mice with head protection the number of GFP positive cells infiltrated into the brain was very small compared to those with whole body irradiation. However even under head protection, PS stimulated the migration of GFP positive cells in the PVN, those were positive for Iba-1. Therefore the results show that chronic PS stimulates accumulation of bone marrow-derived microglia in the PVN. 

Bone marrow-derived microglia from mice with chronic PS-loaded and sham-treated mice have characteristics of CCR2^+^CX_3_CR1^low^ cells that are distinct from CCR2^−^CX_3_CR1^high^ resident microglia. This finding is consistent with a previous study which characterized bone marrow-derived cells infiltrating into the CNS in cases of EAE or CNS injury as Ly-6C^high^CCR2^+^CX_3_CR1^low^ cells [4,7]. 

To isolate both bone marrow-derived microglia and resident microglia, we sorted CD11b^+^ and CD45^low^ cells; therefore, sorted cells were distinct from the CD11b^+^CD45^high^ perivascular macrophages, meningeal macrophages, resident monocytes or inflammatory monocytes [19]. Peripheral blood monocytes are classified into two subtypes, the inflammatory CD11b^+^CX_3_CR1^low^CCR2^+^ M1 monocytes, and the resident CD11b^+^CX_3_CR1^high^CCR2^−^ M2 monocytes [22]. According to chemokine receptor expression, bone marrow-derived microglia in the present study possess M1 monocyte characteristics, while resident microglia of the present study possess M2 monocyte characteristics. Previous reports showed that M1 monocytes activated by brain inflammation or brain injury secrete the pro-inflammatory cytokines TNF-α and IL-1β [23,24]. In the present study, bone marrow-derived microglia aggregated in the PVN expressed higher levels of IL-1β but lower levels of TNF-α compared with resident microglia. 

Recently, several microglia phenotypes have been proposed in Alzheimer’s disease, including M1, M2a, and M2c [25]. M1 represents ‘classically activated’ microglia that participate in inflammatory responses and were derived from “surveying microglia” by stimulation with TNF-α, IL-1β, and IL-6 [25]. M2a and M2c are ‘alternative activated’ microglia, which attenuate inflammatory responses and promote repair of tissue injury [25]. In Parkinson’s disease, other subsets of microglia have been proposed, including classically activated microglia, chronically activated microglia, reactive microglia, and homeostatic microglia. The latter convert to classically activated microglia following acute inflammation, but convert to reactive microglia when expression of inflammatory cytokines is low, and chronic activated microglia when inflammation is prolonged [26]. As shown in previous studies, activated microglia can exert opposite effects on neurodegenerative reactions, for example, microbial pathogens may induce pro-inflammatory effects via toll-like receptors, while anti-inflammatory effects can be induced by apoptotic cells via the phagocytic receptor P2Y6 or the triggering receptor TREM2 [27]. In the present study, bone marrow-derived microglia in the hypothalamus resemble classically activated microglia because of their high expression of IL-1β, but there was no difference in morphology between bone marrow-derived and resident microglia, and their ramified shape matches that of surveying microglia. Therefore they are considered to be an alternative type of microglia from those previously classified. 

The MCP-1/CCR2 chemokine axis is an important mediator of the migration of monocytes, memory T lymphocytes, and natural killer cells into affected areas in diseases such as multiple sclerosis, rheumatoid arthritis, type 2 diabetes, and Alzheimer’s disease [28,29]. Our results show that chronic psychological stress stimulates the production of MCP-1 protein in PVN neurons and increases the mRNA expression of MCP-1 in the hypothalamus. Because bone marrow-derived cells express higher levels of the MCP-1 receptor CCR2 than resident microglia, they migrate into the PVN by the MCP-1/CCR2 axis. Indeed, aggregation of bone marrow-derived microglia in the PVN was blocked by peripheral administration of a CCR2 antagonist. Furthermore, a CCR2 antagonist was demonstrated to improve the anxiety-like behavior caused by chronic PS. Because these mice were not received irradiation and not received bone marrow transplantation, the MCP-1/CCR2 axis in the brain was involved in the mechanism of the abnormal behavior induced by chronic PS. 

The recruitment of bone marrow cells into the peripheral circulation is regulated by the SDF-1/CXCR4 interaction between bone marrow niche cells and hematopoietic stem cells [30–32]. A recent study showed that circadian oscillation originating from the suprachiasmatic nucleus in the brain stimulates β_3_-adrenergic receptors on bone marrow stromal cells via sympathetic pathways [32]. Activation of β_3_-adrenergic receptors on bone marrow stromal cells induces the reduction of SDF-1 production in the stromal cells, resulting in the decrease in the concentration of SDF-1 in the bone marrow. In the special microenvironments, niche, some stromal cells maintain the proliferation and differentiation of hematopoietic stem cells, which express CXCR4 on their surfaces. The SDF-1/CXCR4 interaction is an important axis for the hematopoiesis. Therefore, the circadian oscillation stimulates the shedding of the differentiated cells from hematopoietic stem cells from the bone marrow and then increases the number of the differentiated cells in the peripheral blood [33]. In the present study, we showed that chronic psychological stress decreases the concentration of SDF-1 in bone marrow cells and increases the frequency of CXCR4^+^ monocytes in the peripheral blood; furthermore β_3_-adrenergic inhibition blocked the aggregation of bone marrow-derived cells in the PVN. These results suggest that chronic psychological stress stimulates sympathetic pathways and that activation of β_3_-adrenergic receptors on the bone marrow cells induces a reduction in SDF-1 expression. This might then accelerate recruitment of monocytes from the bone marrow into peripheral circulations. Bone marrow niche cells constitute osteoblastic niche on endosteum and/or vascular niche on sinusoid, which regulate hematopoietic stem cells in differentiation and their recruitment into circulation [31,32]. Therefore chronic psychological stress may affect bone marrow niche functions via above mentioned pathways.

Bone marrow-derived microglia shown here highly express the pro-inflammatory cytokine IL-1β, which has been reported to be a key mediator in the alteration of synaptic signal transmission during injury, infection and diseases of the CNS [34,35]. In the spinal cord, microglia and astrocytes are activated by glutamate, substance P or ATP released from injured afferent presynaptic neurons [34]. These activated glia release various mediators including IL-1β and TNF-α and subsequently depolarize glutamatergic and GABAergic postsynaptic neurons [35]. In the hippocampus, on the other hand, IL-1β enhances both calcium signaling and excitatory postsynaptic currents through phosphorylation of NMDARs , which consists of glutamate-gated ion channels, via IL-1R; these pathways affect neuronal functions in neurodegenerative diseases [36–38] . The present study showed that bone marrow-derived microglia from chronic psychological stress-loaded mice exist adjacent to neurons expressing pNMDARs and IL-1Rs. This indicates that bone marrow-derived microglia control neuronal transmission in PVN via activating phosphorylation of NMDARs through IL-1Rs under chronic PS conditions. IL-1β increases the permeability of blood-brain barrier and leads to the infiltration of leukocytes, macrophages and dendritic cells into brain parenchyma [39]. IL-1β expressing bone marrow derived monocyte could infiltrate into PVN.

The purinergic receptors P2X4, P2X7, P2Y6, and P2Y12 expressed on microglia are also involved in pain signaling in the spinal cord [40]. These receptors are activated by ATP, ADP or UDP released from injured primary afferent neurons, and activated microglia release PGE2, BDNF, TNF-α or IL-1β, which enhances depolarization of primary afferent neurons as well as inhibitory interneurons leading to neuropathic pain [40]. Microglia derived from P2Y12^−/−^ mice were previously found to have no chemotaxic ability toward ATP and ADP [41]. The present results show that expression levels of P2X4, P2X7, P2Y6 and P2Y12 on bone marrow-derived microglia were similar or lower than those of resident microglia, suggesting that these purinergic pathways are not involved in the migration of microglia into specific brain nuclei. 

**Figure 6:**
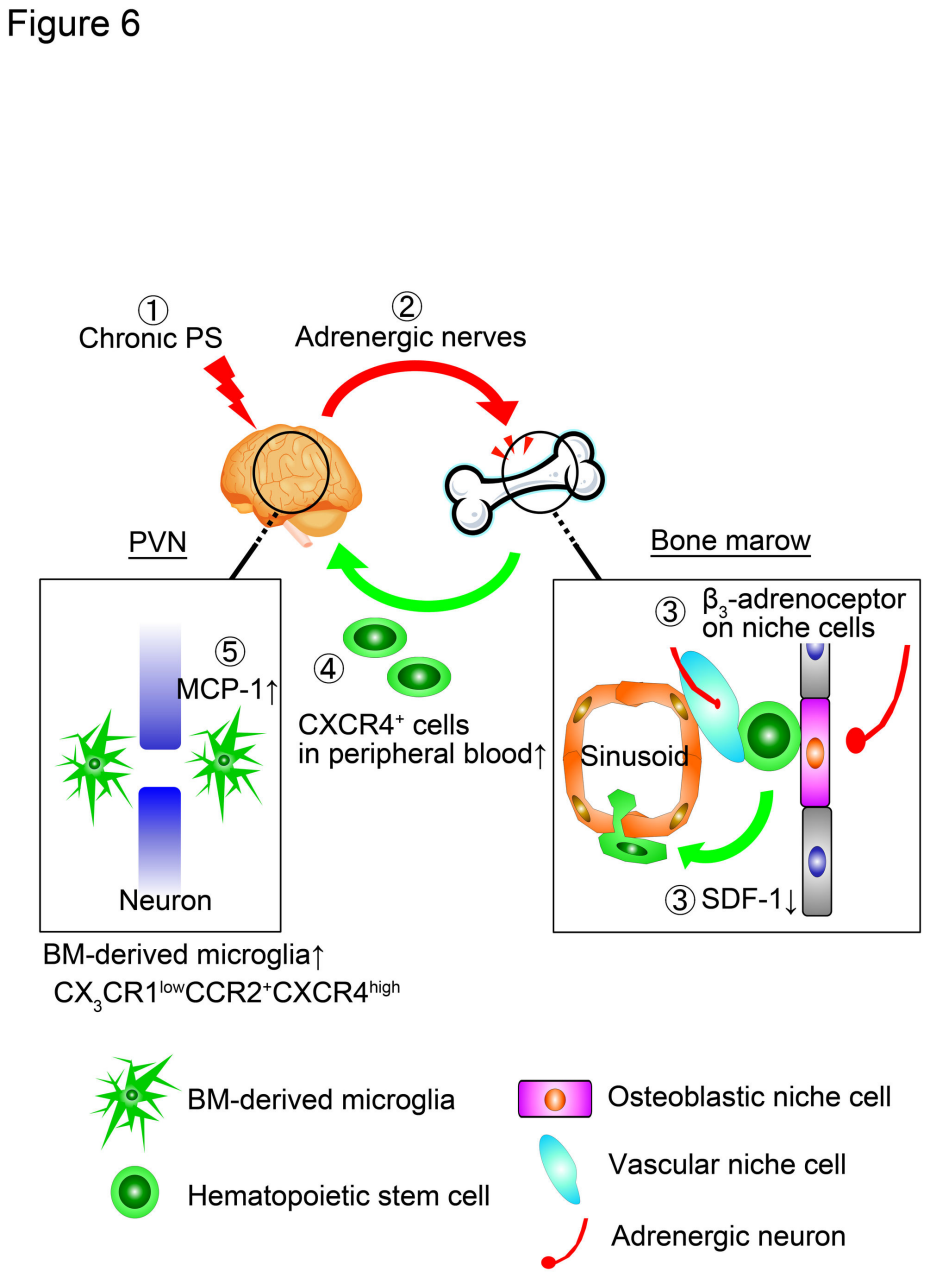
Schematic drawing of brain-bone marrow axis in chronic psychological stress conditions. When brain is exposed to chronic PS (1), the information is mediated to bone marrow through adrenergic nerves (2). Bone marrow niche cells innervated with sympathetic nerves decrease the expression of SDF-1 through β_3_-adrenergic receptor (3). CXCR4^high^ monocytes egress to peripheral circulation from the bone marrow by reduction of SDF-1 at the bone marrow niche (4). Neurons in the PVN express MCP-1 under chronic PS condition, then accelerate infiltration of CCR2^+^ bone marrow-derived microglia into PVN (5).

In conclusion, we demonstrate that chronic psychological stress induces the aggregation of bone marrow-derived microglia in the PVN of mice and stimulates their recruitment into the circulation via the activation of β_3_-adrenergic pathways and a subsequent reduction in SDF-1 expression on bone marrow niche cells (Figure 6). It is conceivable that bone marrow-derived microglia regulate neuronal transmission in the PVN as they attach to pNMDA receptor- or IL-1 receptor-expressing neurons. 

## Supporting Information

Figure S1 **Communication box and chimeric ratio of peripheral blood from mice** (A) Mice with bone marrow transplantation from GFP-Tg donors were placed in PS compartments and witnessed mice in the ES compartment receiving an electrical foot shock. (B, C) Chimeric ratio of mice received whole body irradiation (B) and specific body irradiation with head protection (C).
(TIF)

Figure S2 **Representations of other area in brain of chronic psychological stress-loaded and sham mice.** There are no differences of GFP^+^ cells locations in other areas between the chronic psychological stress-loaded and the sham mouse.
(TIF)

Figure S3 **The number of GFP^+^ cells in hippocampus and central amygdala of chronic psychological stress-loaded and sham mice.** We counted the number of GFP-positive cells in one side of the hippocampus (A) and central amygdala (B) within five successive sections at 200× magnification using confocal laser microscopy, and the maximum number of GFP^+^ cells in a section was the representative data. Hippocampus and central amygdala were distinguished according to Mouse Brain in Stereotaxic Coordinates written by Franklin & Paxinos. In the amygdala the numbers of GFP^+^ cells were counted in the area for 500 µm around of central amygdala.
(TIF)

Table S1 **Primers for quantitative RT-PCR.**
(DOCX)
