# Supplementary figures and images for: Bone Marrow-Derived Microglia Infiltrate into the Paraventricular Nucleus of Chronic Psychological Stress-Loaded Mice
Source: PLoS One. 2013 Nov 26;8(11):e81744. doi: 10.1371/journal.pone.0081744 (PMC3841189; doi:10.1371/journal.pone.0081744)

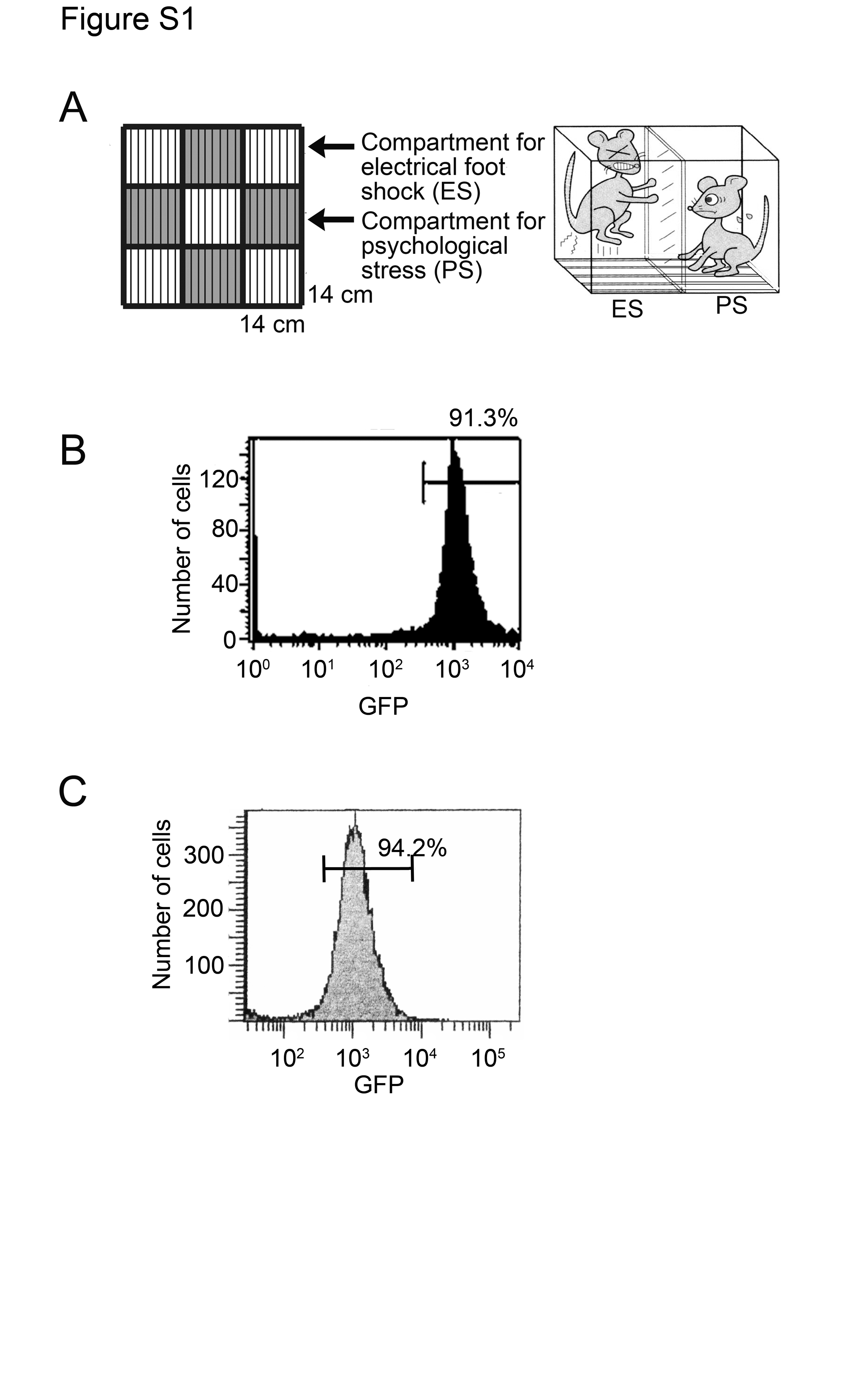

Supplement: Figure S1 — Communication box and chimeric ratio of peripheral blood from mice (A) Mice with bone marrow transplantation from GFP-Tg donors were placed in PS compartments and witnessed mice in the ES compartment receiving an electrical foot shock. (B, C) Chimeric ratio of mice received whole body irradiation (B) and specific body irradiation with head protection (C). (TIF) [file pone.0081744.s001.tif]

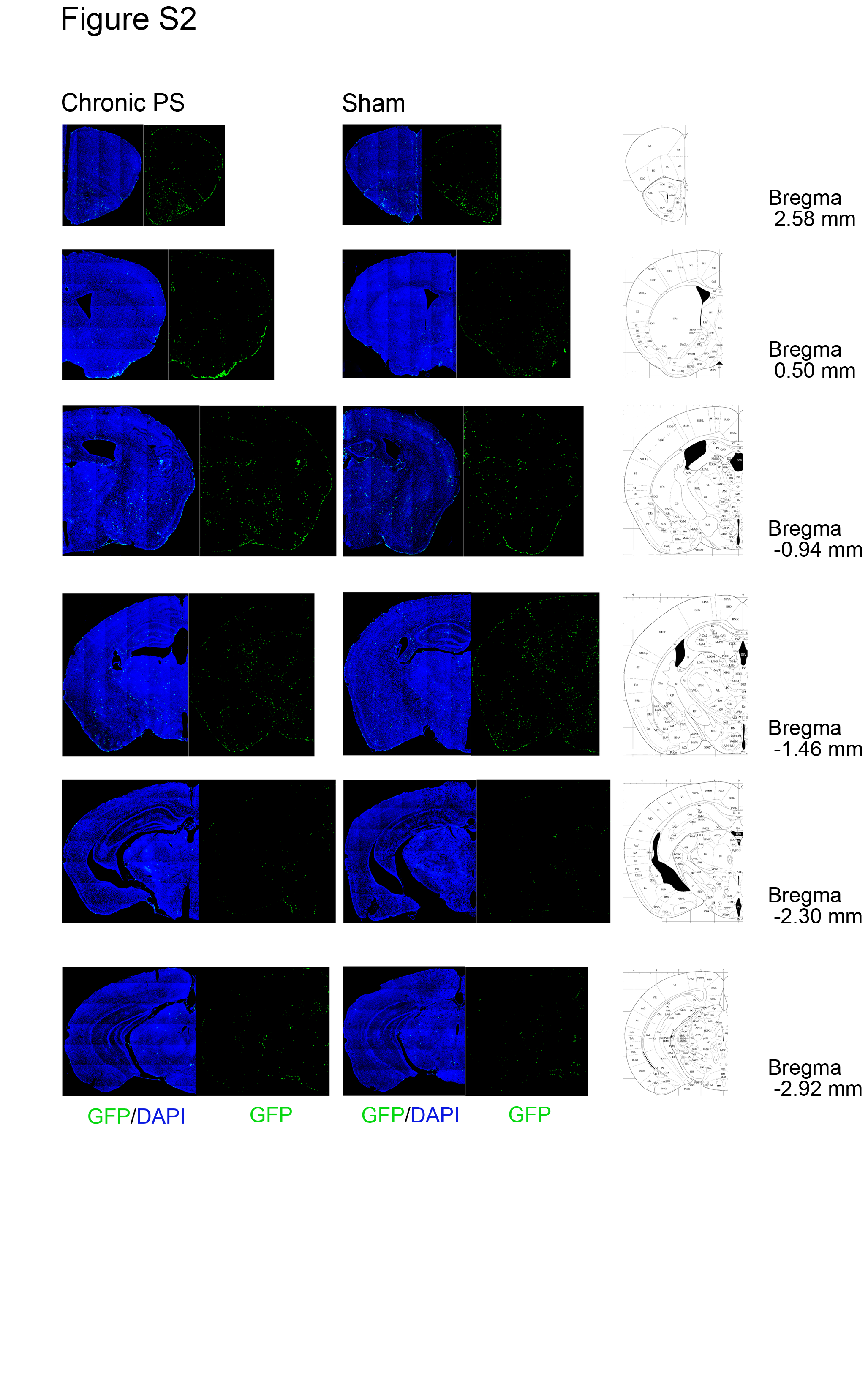

Supplement: Figure S2 — Representations of other area in brain of chronic psychological stress-loaded and sham mice. There are no differences of GFP+ cells locations in other areas between the chronic psychological stress-loaded and the sham mouse. (TIF) [file pone.0081744.s002.tif]

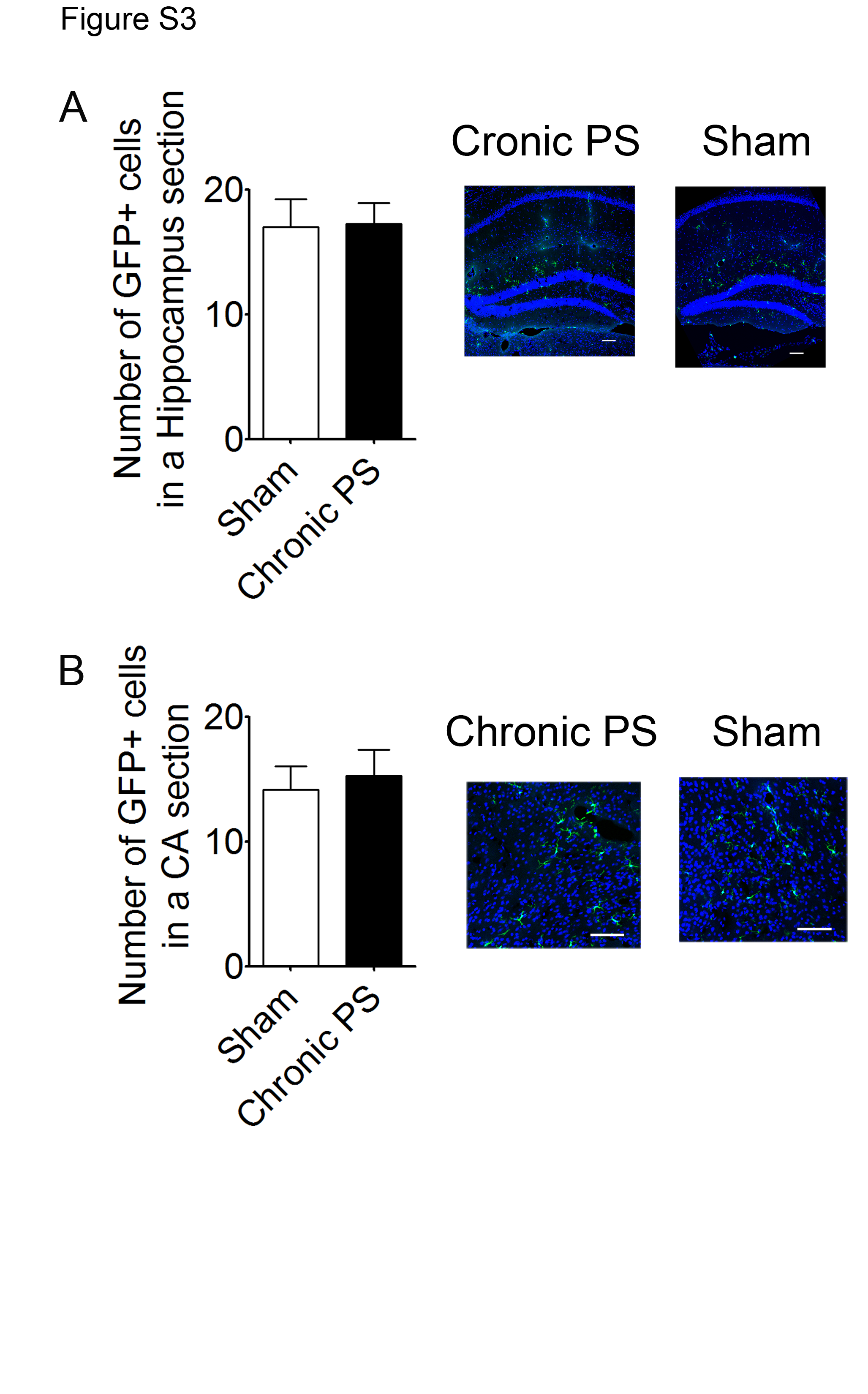

Supplement: Figure S3 — The number of GFP+ cells in hippocampus and central amygdala of chronic psychological stress-loaded and sham mice. We counted the number of GFP-positive cells in one side of the hippocampus (A) and central amygdala (B) within five successive sections at 200× magnification using confocal laser microscopy, and the maximum number of GFP+ cells in a section was the representative data. Hippocampus and central amygdala were distinguished according to Mouse Brain in Stereotaxic Coordinates written by Franklin & Paxinos. In the amygdala the numbers of GFP+ cells were counted in the area for 500 µm around of central amygdala. (TIF) [file pone.0081744.s003.tif]
