# Supplementary material for: Bone Marrow-Derived Microglia Infiltrate into the Paraventricular Nucleus of Chronic Psychological Stress-Loaded Mice
Source: PLoS One. 2013 Nov 26;8(11):e81744. doi: 10.1371/journal.pone.0081744 (PMC3841189; doi:10.1371/journal.pone.0081744)
Supplement: Table S1 — Primers for quantitative RT-PCR. (DOCX) [file pone.0081744.s004.docx]

Table S1.　Primers for quantitative RT-PCR

| Gene | NM number | Forward primer | Reverse primer |
| --- | --- | --- | --- |
| CCR2 | NM_009915 | gatgtcatgtgtgcatgcaagt | ttcccactcactcaaaggacaa |
| CX_3_CR1 | NM_009987 | ctccctgttcctgtgttg gttg | aaagggttggatatgcctgtgg |
| CXCR4 | NM_009911 | tgctgtgtgatggtttgtttgg | aaaacccccagcatttctacca |
| EAAT1 | NM_148938 | tgccatagacccacagcatatc | cctgtctgagatcctcatggtg |
| EAAT2 | NM_001077514 | cctccctttcctcacagatcat | catccataatttgaagggcaca |
| P2X4 | NM_011026 | CGTGCTGTGTGACGTCATAGTC | GGAAAGACCCTGCTCGTAGTCT |
| P2X7 | NM_001038839 | agccgacgttgaagtatgtgtc | aggttggaacttcttggccttt |
| P2Y1 | NM_008772 | GCAGAATGGAGACACGAGTTTG | CTCAGGGATGTCTTGTGACCAT |
| P2Y12 | NM_027571 | TCCCGGAGACACTCATATCCTT | GAGAAGGTGGTATTGGCTGAGG |
| IL-1β | NM_008361 | CAGGATGAGGACATGAGCACC | CTCTGCAGACTCAAACTCCAC |
| TNF-α | NM_013693 | CACGTCGTAGCAAACCACCAAGTG | GATAGCAAATCGGCTGACGGTGTGG |
| GAPDH | NM_017008 | TGACTCTACCCACGGCAAGTT | GATGGGTTTCCCGTTGATGA |
